# Effect of a multi-faceted quality improvement intervention on inappropriate antibiotic use in children with non-bloody diarrhoea admitted to district hospitals in Kenya

**DOI:** 10.1186/1471-2431-11-109

**Published:** 2011-11-25

**Authors:** Charles Opondo, Philip Ayieko, Stephen Ntoburi, John Wagai, Newton Opiyo, Grace Irimu, Elizabeth Allen, James Carpenter, Mike English

**Affiliations:** 1 Child and Newborn Health Group, KEMRI-Wellcome Trust Research Programme, Nairobi, Kenya; 2 Department of Paediatrics and Child Health, University of Nairobi, Nairobi, Kenya; 3 Department of Medical Statistics, London School of Hygiene and Tropical Medicine, London, UK; 4 Department of Paediatrics, University of Oxford and John Radcliffe Hospital, Headington, Oxford, UK

## Abstract

**Background:**

There are few reports of interventions to reduce the common but irrational use of antibiotics for acute non-bloody diarrhoea amongst hospitalised children in low-income settings. We undertook a secondary analysis of data from an intervention comprising training of health workers, facilitation, supervision and face-to-face feedback, to assess whether it reduced inappropriate use of antibiotics in children with non-bloody diarrhoea and no co-morbidities requiring antibiotics, compared to a partial intervention comprising didactic training and written feedback only. This outcome was not a pre-specified end-point of the main trial.

**Methods:**

Repeated cross-sectional survey data from a cluster-randomised controlled trial of an intervention to improve management of common childhood illnesses in Kenya were used to describe the prevalence of inappropriate antibiotic use in a 7-day period in children aged 2-59 months with acute non-bloody diarrhoea. Logistic regression models with random effects for hospital were then used to identify patient and clinician level factors associated with inappropriate antibiotic use and to assess the effect of the intervention.

**Results:**

9, 459 admission records of children were reviewed for this outcome. Of these, 4, 232 (44.7%) were diagnosed with diarrhoea, with 130 of these being bloody (dysentery) therefore requiring antibiotics. 1, 160 children had non-bloody diarrhoea and no co-morbidities requiring antibiotics-these were the focus of the analysis. 750 (64.7%) of them received antibiotics inappropriately, 313 of these being in the intervention hospitals vs. 437 in the controls. The adjusted logistic regression model showed the baseline-adjusted odds of inappropriate antibiotic prescription to children admitted to the intervention hospitals was 0.30 times that in the control hospitals (95%CI 0.09-1.02).

**Conclusion:**

We found some evidence that the multi-faceted, sustained intervention described in this paper led to a reduction in the inappropriate use of antibiotics in treating children with non-bloody diarrhoea.

**Trial registration:**

International Standard Randomised Controlled Trial Number Register ISRCTN42996612

## Background

Diarrhoea remains one of the leading causes of mortality in childhood, accounting for 15% of the approximately 8.7 million deaths of under-5 year olds worldwide in 2008 alone [1]. Current best-practice guidance for treatment of diarrhoea is contained within the World Health Organisation's strategy for Integrated Management of Childhood Illness (IMCI), a strategy adopted by over 100 countries worldwide [2]. This strategy is intended to foster correct diagnosis and treatment of common childhood illnesses in outpatient and inpatient settings with guidance for small hospitals published in the Pocket Book of Hospital Care for Children [3]. However reports indicate that success in implementation of diarrhoea case management has been varied [4-8].

Treatment guidelines for diarrhoea emphasise that patients be assessed for presence of blood in stool; non-bloody diarrhoea is to be managed with fluids only (unless co-morbidities are present which require different treatment), while bloody diarrhoea, presumed to be dysentery, should be managed with fluids and antibiotics. This is a pragmatic approach informed by the observation that most non-bloody diarrhoea episodes in the under-5 age group in low-income settings are self-limiting and are caused by pathogens not susceptible (e.g. rotavirus, astrovirus and enteric adenovirus) to antibiotic therapy or for which antibiotics are likely of little value or even deleterious (e.g. salmonellae and campylobacter) [9]. In contrast a significant proportion of episodes of bloody diarrhoea caused by shigella are associated with considerable mortality and are ameliorated by antibiotic therapy [10]. Furthermore correcting dehydration is clinically more important due to its association with adverse outcomes. Despite the fact that the guidelines are unambiguous, inappropriate antibiotic use remains common [11]. Antibiotic misuse may accelerate acquisition of antibiotic resistance [12] necessitating the use of more expensive alternative drugs [13,14] and is a serious threat to drug therapy of infectious diseases [15,16]. It may also be associated with missed opportunities to make correct diagnoses and as a result further increase in treatment cost. These undesirable consequences are disproportionately greater in low income countries which bear a bigger burden of infectious diseases [17]. However, research that explores the reasons for unnecessary antibiotic use in low-income settings is unusual; research that explores the effects of interventions on this outcome, particularly amongst inpatient populations, is rare. We conducted a multi-faceted intervention trial in Kenya aimed at improving key inpatient paediatric care practices spanning assessment, diagnosis and treatment of malaria, pneumonia and non-bloody diarrhoea using 14 process-of-care indicators. The effect of this intervention on these practices has been reported previously [18]. Here, we use these data to investigate whether the intervention (described subsequently) reduces inappropriate antibiotic use in the children admitted with non-bloody diarrhoea using a hierarchical modelling approach.

## Objective

This trial was undertaken between 2006 and 2009 with the main end-point in 2008. It investigated whether an intervention delivered at the hospital level comprising training health workers on the use of evidence-based guidelines for paediatric care, local facilitation, and external supervision and feedback, would improve the quality of care given to children with specific diagnoses admitted to Kenya's district hospitals [18]. In this paper we explore associations between the intervention and evidence for inappropriate antibiotic use in cases of non-bloody diarrhoea. This outcome was not a pre-specified trial endpoint.

## Methods

### Setting

Kenya has a population of just over 39 million (2009 population census). Infant mortality rate is 54 deaths per 1000 live births and under-five mortality rate is 78 per 1000. Diarrhoea causes 20% of under-five deaths [19]. Administratively, Kenya has eight provinces each subdivided into districts; in total there were 70 districts (1999 boundary review) at the time of the study each with a district hospital providing walk-in and referral care. This study was conducted in eight district hospitals purposefully selected to represent typical variations in disease risk and hospital size from four of the eight provinces [20]. A restricted randomisation procedure was then used to assign the 8 hospitals into two groups of four in an effort to maintain balance across the groups with respect to the key characteristics considered during selection [18].

Due to the nature of the intervention it was impossible to blind the health workers or the research team delivering the intervention to the group allocations. However the caretakers of children whose case records were examined were unaware of these allocations. The admission records of children aged 1 day to 12 years hospitalised with a diagnosis of one or more of the common serious childhood illnesses including diarrhoea, dehydration, malaria, pneumonia, meningitis, malnutrition, anaemia and neonatal sepsis were selected using a random and uniform sample of admission dates between the 6-monthly surveys.

### The intervention

A detailed description of the development and context of the intervention is available elsewhere [20,21]. Briefly, it involved adaptation of evidence-based practices for assessing, classifying and managing childhood malaria, pneumonia, diarrhoea and dehydration, malnutrition, anaemia, meningitis, neonatal sepsis and prematurity to the local situation with development of management protocols disseminated by the Ministry of Health. Thus, training spanned a large number of recommendations. Recommendations for managing non-bloody diarrhoea alone included appropriate history-taking, assessment for shock and dehydration, classification of severity of dehydration and appropriate rehydration therapy, four key recommendations (of which one was a primary trial outcome-accuracy of intravenous fluid prescriptions) [18]. The use of antibiotics for non-bloody diarrhoea was actively discouraged and represented a fifth key recommendation in this area. This management plan was summarised within the clinical protocols provided within guideline booklets for clinicians and nurses and wall charts.

A training programme for health workers was developed to help implement these guidelines (called ETAT+, described in full by Irimu *et al*. [22]) and piloted prior to their use in this study. The full intervention had several components including: the five-and-a-half day theory and practical training (ETAT+) in recommended practices for 30-34 hospital staff at each intervention site (in total 90 nurses, 11 medical officers and 29 clinical officers providing paediatric care referred to here as 'clinicians' were trained across the four hospitals); clinical practice guidelines and job-aids for all health workers providing paediatric care; 2-3 monthly supervision of hospitals' implementation by a paediatrician from the study team to discuss progress in guideline implementation, informed by data from surveys when available (see below), and identify local strategies for problem-solving with senior hospital staff (described by Nzinga *et al*. [23]); a local facilitator from among the hospital staff assigned to promote guideline implementation for 18 months; six-monthly surveys to assess progress and collect data for the study; and written and face-to-face feedback on findings after each survey. Written and face to face feedback focused on key trial outcomes but also provided information on inappropriate antibiotic use for non-bloody diarrhoea amongst many other indicators of quality of care. Face to face feedback was provided by those undertaking supervision six-monthly, however the wider supervisory process (2-3 monthly) was not standardised tending to focus on key, hospital specific problems related to pre-identified key indicators [18] and follow up of locally developed action plans. While the main trial outcomes were thus the focus of intervention it was hoped that by providing a broad set of guidelines, linked to training and an increased emphasis on provision of quality care fostered by the process of supervision and feedback that a wider set of practices might improve.

A partial intervention was delivered to 'control' hospitals including: the same 1.5 days didactic training on the use of guidelines as in the full 5.5 days course that was given to 35-40 hospital staff at each site (107 nurses, 6 medical officers and 21 clinical officers in total across the four sites); the clinical practice guidelines and job-aids; and written feedback after contemporaneous surveys. The partial intervention was not typical of this setting; it was better than the support that would routinely have been provided by the Ministry of Health, notably in the provision of written performance feedback.

The study began with baseline surveys in July and August 2006 conducted over two weeks at each hospital. Subsequent surveys were done in 6-month intervals over the next 18 months during which the intervention was delivered. The main outcome for this analysis was inappropriate prescription of antibiotics to children with non-bloody diarrhoea.

### Data collection procedures and tools

During each survey records of patients admitted to hospital with acute illness on a set of random dates within the preceding six months (generated using Stata™) were selected with the aim of obtaining 400 records per hospital per survey [18]. Each record was assigned a unique ID number linking to a hospital and survey, and another ID number for the attending clinician linking to clinicians' characteristics. Data were collected using a patient case-record data abstraction form. Data collectors were trained for three weeks prior to the first data collection exercise and sent to the field in four teams of four each supervised by a member of the study team [20]. It was not possible to blind the data collectors to the group allocations. However the objectivity and consistency of the collected data was improved by rotating the teams for subsequent data collection exercises. 10% of the data at each site was double-collected during each survey to assess agreement which was consistently above 95%.

## Data

### Eligible sample

**Figure 1:**
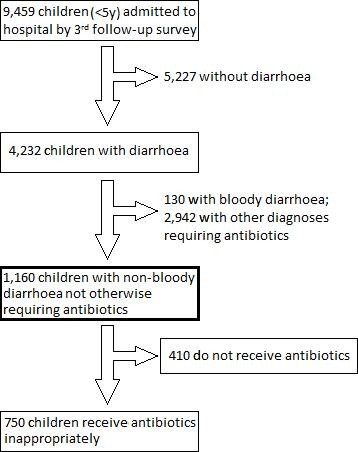
**Sample profile; the bold rectangle shows the group of children of interest to this analysis**.

By the study end-point (survey 3) 9, 459 admission records of under-5 year olds were available for review. Of these, 5, 358 (56.6%) children had diagnoses requiring antibiotic therapy according to guideline recommendations. 4, 232 (45.7%) were diagnosed with diarrhoea but only 1, 160 (median across hospitals 147.5, range 53-227) cases were non-bloody and did not require antibiotics for an additional (co-morbid) diagnosis. Of these 750 (64.7%) (313 in intervention group and 437 in control group) received antibiotics inappropriately; this is the outcome of interest (Figure 1).

### Exposures and outcome measures

**Figure 2:**
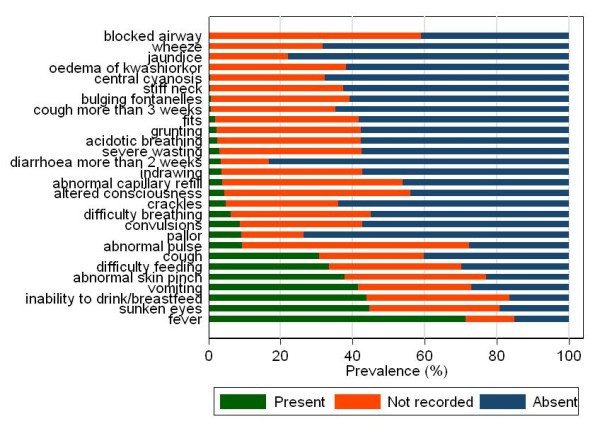
**Prevalences of clinical signs among the 1, 160 children with diarrhoea**.

Table 1 summarises key characteristics of the 1, 160 children with non-bloody diarrhoea while Figure 2 illustrates the prevalences of the various clinical signs observed among them. 308 clinicians were responsible for admitting these children. The number of admissions by each clinician varied widely, from 96 different clinicians admitting one child each, to one clinician admitting 27 children over the 24 month period (median admissions per clinician: 3). There were 229 clinicians responsible for inappropriate antibiotic prescriptions ranging from 96 clinicians prescribing to one child each, to one clinician responsible for prescription to 22 children (median prescriptions per clinician: 2). Key characteristics of the 308 clinicians are summarised in table 2.

**Table 1:** Key characteristics of the 1, 160 children with non-bloody diarrhoea

Age (years)	Median 0.8, IQR 0.6-1.3, N = 1, 160
**Gender**	Male 550 (47.4%); female 441 (38.0%); not recorded 169 (11.3%), N = 1, 160

**Weight (kg)**	Median 8.0, IQR 6.6-9.0, N (recorded) = 728

**Length of illness prior to admission (days)**	Median 3.0, IQR 2.0-5.0, N (recorded) = 1, 062

**Table 2:** Summary of characteristics of clinicians admitting the 1, 160 children (CO = clinical officer, MO = medical officer, paed = paediatrician)

Gender		Qualifications		Age group (years)		Experience (years)	
Male	134 (43.5%)	CO intern	107 (34.7%)	20-24	56 (18.2%)	< 1	140 (45.5%)
Female	78 (25.3%)	CO	58 (18.8%)	25-29	112 (36.4%)	1-5	37 (12.0%)
Unknown	96 (31.2)	MO	45 (14.6%)	30-34	23 (7.5%)	6-10	12 (3.9%)
		Paed.	1 (0.3%)	35-39	10 (3.3%)	> 10	8 (2.6%)
		Unknown	97 (31.5%)	40-44	3 (1.0%)	Unknown	111 (36.0%)
				45-49	2 (0.7%)		
				50-54	1 (0.3%)		
				55-59	1 (0.3%)		
				Unknown	100 (32.5%)		

**Figure 3:**
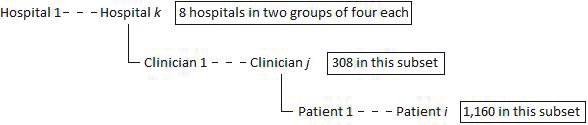
**Hierarchical structure of the data**.

It is important to note that every hospital had several clinicians each of whom attended to one or more children whose records we reviewed; this leads to a hierarchical data structure which will need to be accounted for in all analyses (Figure 3). The outcome (described previously) is a binary variable derived from the patient's clinical record at admission representing whether the use of antibiotics based on the child's diagnosis was appropriate or not. Children with non-bloody diarrhoea who received any antibiotic but neither had pneumonia, malnutrition, meningitis, neonatal sepsis nor any other bacterial infection, were coded as having received antibiotics inappropriately as this is contrary to advice in national guidelines.

### Statistical analysis

All children were assumed to have received the group treatment their hospitals had been allocated to therefore the analyses described here adhere to the 'intention-to-treat' principle. The trial was a cluster randomised trial, and any analysis needs to allow for this. Given that individual level regression methods do not necessarily perform robustly when there are relatively few clusters [24], we adopted a two stage approach to the analysis [25]. The first step was to carry out an unadjusted cluster-level analysis where we calculated the observed proportions of children receiving inappropriate antibiotics at each hospital during each survey. The difference in the mean proportions in the control and intervention groups was then assessed using a t-test. An adjusted cluster-level analysis was then undertaken. A forward-and-backward stepwise variable selection procedure within a hierarchical logistic regression model was used to identify predictors of inappropriate antibiotic use from the patient-level factors in table 1 and Figure 2 and clinician-level factors in table 2. We then fit a logistic regression model using as covariates the factors found to be associated with the outcome excluding the effect we are interested in (here the effect of the intervention). We then obtained covariate-adjusted residuals based on the difference between observed proportions of children experiencing the outcome at baseline and the main end-point versus predicted proportions from the logistic model. This *difference-residual *was computed for each cluster (hospital) and difference between the mean difference-residual in the control and intervention groups was again assessed using a t-test. In the absence of an effect the group means of these residuals should be similar.

The second approach to the analysis was a multilevel logistic regression model to estimate the intervention effect. Details of this model are given in the appendix. The model was adjusted for survey period and other previously identified important covariates. It also included interaction terms to estimate how the effect of the intervention was modified by the different survey periods thus allowing for time. Likelihood ratio tests were carried out to decide which terms to retain in the model. Hospital and clinician intra-cluster correlation coefficients were estimated from the final model [26].

All analyses were done using Stata™ v.11 (Stata Corp., College Station, Texas) and were based on complete case-records only. Ethical approval for the trial was granted by Kenya Medical Research Institute National Ethics and Scientific review committees (SSC No. 991).

## Results

**Figure 4:**
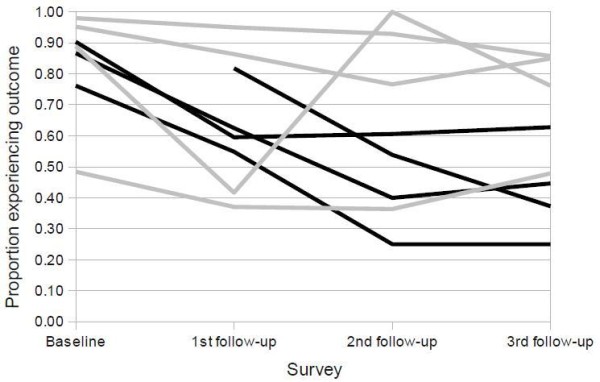
**Proportions of children receiving inappropriate antibiotics; black and grey lines represent intervention and control hospitals respectively**.

There was a marked decline in the proportions of children receiving antibiotics inappropriately in the intervention hospitals between baseline and the main study end-point (Figure 4). A similar but more modest trend was observed in the control hospitals (at baseline in one intervention hospital, H2, none of the children admitted met the criteria for inclusion into this sample). We observed an important unadjusted difference between the mean proportions of children receiving inappropriate antibiotics at the main end-point in the intervention versus control hospitals (0.42 vs. 0.74 respectively, p-value 0.04). However the adjusted difference in proportions was 0.41 (95%CI -0.06 to 0.88, p-value 0.077) providing only weak evidence of an intervention effect. In the individual level analysis crackles (an auscultated clinical sign often associated with lower respiratory tract infection) was found to be independently associated with the outcome and included in the adjusted model. Table 3 summarises the final multilevel logistic regression model. The *adjusted *odds of the outcome in the intervention group at the main end-point of the study (3^rd ^follow-up survey) was 0.30 times that in the control group (95%CI 0.09-1.02). Hospital and clinician-level intra-cluster correlation coefficients were 0.17 and 0.68 respectively suggesting that there was greater homogeneity of the outcome in children attended to by the same clinician, than those visiting the same hospital. Model diagnostics confirmed the appropriateness of fit of this model (see the additional file 1).

**Table 3:** The multilevel logistic regression model with clinician and hospital random effects

Variable	N	Levels	Odds ratio	95% CI	p-value
Group	566	Control			0.843
	594	Intervention	0.85	0.17-4.30	

Survey	275	Baseline			0.159
	222	1^st ^Follow-up	0.37	0.14-0.96	
	220	2^nd ^Follow-up	0.42	0.16-1.16	
	443	3^rd ^Follow-up	0.41	0.17-0.98	

Group × Survey	103	Intervention × Baseline			0.048
	119	Intervention × 1^st ^Follow-up	1.03	0.28-3.72	
	109	Intervention × 2^nd^Follow-up	0.30	0.08-1.15	
	263	Intervention × 3^rd ^Follow-up	0.30	0.09-1.02	

Crackles	742	Absent			< 0.001
	56	Present	6.98	2.61-18.67	
	362	Not recorded	2.11	1.29-3.44	

**Random-effects**	**N**	**Component**	**Estimate**	**95% CI**	
Clinician	308	Standard deviation	1.20	0.92-1.57	
Hospital	8	Standard deviation	0.90	0.51-1.57	

## Discussion

Clinical practice guidelines are applied in healthcare settings with the aim of improving the process of care. Studies in a variety of settings and clinical fields generally show that guidelines improve processes and outcomes of care [27]. However most of these studies have been poor quality individually randomised studies or studies in primary healthcare settings that are not comparable to hospitals in a low income country. Guidelines are very important when new evidence makes it necessary to change practice. In cases of non-bloody diarrhoea in children guidelines discourage the use of antibiotics. Focusing on this as a process-of-care measure for diarrhoea case management we sought to determine whether this outcome was influenced by an intervention promoting the uptake of a set of practice guidelines aimed at health workers caring for children admitted to hospital. This outcome is relevant to our setting because childhood diarrhoea and dehydration are leading causes of death, and contemporary evidence favours effective supportive treatments such as rehydration while discouraging antibiotic therapy except in cases of dysentery [9]. Furthermore inappropriate use of antibiotics is a waste of important resources that a low-income country can ill afford. While other interventions are also important in reducing the burden of disease and costs attributable to diarrhoea, such as staff hand-washing that was also promoted within the intervention, our study design did not allow us to evaluate any effect of the intervention on these practices.

We used two approaches to the analysis of the data: an adjusted t-test to assess evidence of a difference in mean effect between the full intervention and control (partial intervention) hospitals-a simple but robust approach [25]-and a hierarchical logistic regression model for these repeated survey data. The latter method allows for the clustering within the data in acknowledgement of the reality that the hospital is the unit of intervention, but can be faulted for underestimating higher-level variances for binary covariates when lower-level units (clusters) are few relative to higher-level units (individuals) [24]. Imbalances may also occur between intervention and control groups with respect to covariates predictive of the outcome when the number of higher-level units is relatively small.

Both procedures provide comparable weak evidence of a possibly substantial intervention effect, with the individual level model indicating that children in the intervention hospitals had just under one-third the odds of receiving antibiotics inappropriately compared to those in the control hospitals by the trial endpoint. This evidence of an intervention effect complements that derived from analyses of the trial's main outcomes (structure indicators of availability of key resources supporting service delivery, process errors in management of pneumonia, malaria and diarrhoea/dehydration, and outcome indicators of adherence to key policy recommendations) [18] and suggests broader benefits than so far reported. A new finding was some evidence that the intervention effectiveness was modified by survey period; this suggests a dose-dependent effect on the outcome. Thus the accelerated improvement in the intervention arm compared with the control arm suggests a true effect of the full intervention. For ethical reasons no comparable data are available from hospitals without any form of intervention although baseline data perhaps indicate typical prevalence of inappropriate antibiotic use. It is therefore not possible to determine if the less marked improvement in hospitals receiving the partial intervention was also an intervention effect or represented just a secular trend. Nevertheless this, along with other results of the trial [18], represents the first evaluation of a complex intervention in our setting and evidence that it promotes good paediatric care practice.

Restricted randomisation was used to create the group allocations. It must be acknowledged that when randomising only 8 units of analysis the potential for residual confounding, even after identifying and adjusting for other associations with the outcome as above, is considerable. Still, the model showed no evidence that the baseline differences in the outcome between the hospitals was more than would have been expected by chance (OR 0.85, p-value 0.843). Furthermore the objective nature of the exposures and outcome further strengthen the analysis. Interestingly between-clinician differences contributed almost two-thirds of overall variability in the data, suggesting that hospitals as organisations do not necessarily promote consistent practice amongst clinicians within them.

We found that children with crackles had increased odds of receiving inappropriate antibiotics. Crackles are commonly associated in health worker training with lower respiratory tract infection. However in the national (and international) guidelines provided and used in training the diagnosis of pneumonia, which requires treatment with antibiotics, does not depend on observing crackles. Rather, it should be made in children with elevated respiratory rates or other signs of respiratory distress. Our results and anecdotal experience however suggest clinicians generally find this diagnostic advice difficult to accept. Nevertheless, and interestingly, in these data the clinicians recording the presence of crackles did not record a diagnosis of pneumonia; in the analysis this therefore was classified as inappropriate antibiotic use. Absence of information on crackles was also associated with inappropriate antibiotic use. An alternative or complementary hypothesis is that clinicians who carefully record clinical signs, and find them to be negative, are more likely to adhere to guidelines. Qualitative research might be particularly useful to examine such hypotheses and facilitate understanding of inappropriate antibiotic use in hospitalised children with diarrhoea.

### Limitations of the study

The main limitation from the study design is the relatively small sample of 8 hospitals. This is likely to be insufficient in characterising the diversity of patients, clinicians and hospitals in the country. However feasibility issues make working with large numbers of hospitals difficult in low-income settings [21]. Missing data is another problem, and this is especially true of clinician-level data. This may explain why few clinician-level variables were important in the combined adjusted models. Adjustment for baseline values was an important part of the analysis; however missing and poor quality data was most prevalent at the baseline survey and this may have led to misclassification of patients as not needing antibiotics more often than in subsequent surveys; the effect of this would be to overestimate the true change of the outcome over the course of the intervention.

A limitation arising from the analysis strategy and procedures include the use of the stepwise variable selection procedure, which in performing a large number of tests increases the probability of 'false positive' findings and models based on chance findings [28]. Models constructed using this procedure tend to have very small p-values and may be sensitive to small changes in the underlying data, and may also be poor at predicting outcomes in different situations from those generating the data from which they are based. Another limitation is the fact that this was a post-hoc analysis which was not specifically anticipated in the original study design; the data available from the study may therefore not be sufficient for answering the questions posed.

Finally, patients' signs and symptoms not included in the case record were deemed to have been unavailable to the clinician to help in making a diagnosis and were coded as 'not recorded'. This category obviously contained a mix of patients with and without the sign/symptom. A bias may have arisen if there was an association between the missing observations and the outcome [29]. Multiple imputation can be used to further explore this issue.

## Conclusions

We are not aware of any other studies evaluating an intervention intending to improve care for multiple, common and life-threatening illnesses in children admitted to hospitals in low-income settings using a randomised controlled design. The intervention provided training on evidence-based paediatric care with facilitation, supervision and feedback delivered at the hospital level. In the analyses presented we show that the intervention substantially reduced the odds of a child being inappropriately treated with antibiotics for non-bloody diarrhoea, an improvement in care that is in addition to the findings of improvement in most of the 14 process of care indicators forming the primary endpoint for the trial [18]. Most previous intervention studies to improve care, in both developed and developing countries, have focused on single diseases and have often demonstrated only modest improvements in practices, The data we bow report indicates even wider benefits of the intervention than suggested in the main study results implying that the intervention effects may cut across all diseases covered by the guideline package approach. Other ongoing analyses of the intervention suggest that it requires modest financial investments for comparatively substantial improvements in quality of care. Enhanced medical education focusing on rational use of antibiotics is necessary to improve clinicians' prescribing habits; we have shown that it is possible to change practices using an integrated approach and that perhaps, at hospital level, this is preferable to the recent focus on disease-specific interventions within low-income settings.

## Competing interests

The authors declare that they have no competing interests.

## Authors' contributions

The idea for the main trial was conceived by ME who obtained the funding for this project. CO, SN, JW, NO, PA, GI and ME collected the data. CO, PA, EA and JC contributed to the reported analyses of the data and CO produced the draft manuscript to which all authors contributed during its development. All authors approved the final version of the report.

## Appendix: the hierarchical logistic regression model

Let *Y_ijk _*be the outcome of patient *i *who was attended to by clinician *j *in hospital *k*. Let *x_1 _*be a binary variable representing the intervention group, *x_2 _*a binary variable representing the survey (baseline vs. final post-intervention), and *x_3 _*an interaction parameter to represent how the intervention is modified by successive surveys. We also define *z_l _*as any other covariate(s) associated with the outcome to be adjusted for in the model.

Our model is:

$$\text{E}\left[\text{log it}\left(Y_{ijk}\right)\right]=\alpha +\beta_{1}x_{1ijk}+\beta_{3}{\text{x}}_{3ijk}+\Sigma_{l}\gamma_{l}Z_{ijkl}+u_{j}+u_{jk}$$

with clinician-level random-effects *u_j _*~ N(0, σ*u_j_*^2^), and, conditional on *u_j_*, hospital-level random-effects *u_jk _*~ N(0, σ*u_jk_*^2^).

Our interest is to obtain the estimate of the effect of the intervention on cases examined at main end-point of the study adjusting for the baseline level of the outcome and other important covariates. Consider the form of the model at any survey *S*:

$$\text{E}\left[\text{log it}\left(Y_{ijk}\right)\right]=\alpha +\beta_{1}x_{1ijk}+\beta_{2}S+\beta_{3}Sx_{1ijk}+\Sigma_{l}\gamma_{l}Z_{ijkl}+u_{j}+u_{jk}$$

This can be rearranged to:

$$\text{E}\left[\text{log it}\left(Y_{ijk}\right)\right]=x_{1ijk}\left(\beta_{1}+\beta_{3}S\right)+\alpha +\beta_{2}S+\Sigma_{l}\gamma_{l}Z_{ijkl}+u_{j}+u_{jk}$$

showing that the relationship between the outcome and the intervention has a slope of β*_1 _*+ β*_3_S *and random intercept α + β*_2_S *+ Σ*_l _*γ*_l _z_ijkl _*+ *u_j _*+ *u_jk_*.

Thus, at baseline (*S *= 0) the outcome for a patient in the control group (*x_1 _*= 0) is predicted by:

$$\text{E}\left[\text{log it}\left(Y_{ijk}\right)\right]=\alpha +\Sigma_{l}\gamma_{l}Z_{ijkl}+u_{j}+u_{jk}$$

and:

$$\text{E}\left[\text{log it}\left(Y_{ijk}\right)\right]=\alpha +\beta_{1}x_{1ijk}+\Sigma_{l}\gamma_{l}Z_{ijkl}+u_{j}+u_{jk}$$

in the intervention group (*x_1 _*= 1). The difference between these two patients is the intervention effect at baseline, which is β*_1_*.

Similarly at the main end-point of the study when *S *= *s *the model to predict the outcome for a patient in the control group is:

$$\text{E}\left[\text{log it}\left(Y_{ijk}\right)\right]=\alpha +\beta_{2}s+\Sigma_{l}\gamma_{l}Z_{ijkl}+u_{j}+u_{jk}$$

and:

$$\text{E}\left[\text{log it}\left(Y_{ijk}\right)\right]=x_{1ijk}\left(\beta_{1}+\beta_{3}s\right)+\alpha +\beta_{2}s+\Sigma_{l}\gamma_{l}Z_{ijkl}+u_{j}+u_{jk}$$

in the intervention group. The intervention effect due to this survey is β*_1 _*+ β*_3_s*. To obtain the baseline-adjusted intervention effect we find the difference between effects at these two study points, which is β*_3_s *- the interaction between the intervention and survey *s*.

## Pre-publication history

The pre-publication history for this paper can be accessed here:

http://www.biomedcentral.com/1471-2431/11/109/prepub

## Supplementary Material

Additional file 1 **Hierarchical logistic regression model diagnostics**. Click here for file
