# Supplementary material for: Effect of a multi-faceted quality improvement intervention on inappropriate antibiotic use in children with non-bloody diarrhoea admitted to district hospitals in Kenya
Source: BMC Pediatr. 2011 Nov 25;11:109. doi: 10.1186/1471-2431-11-109 (PMC3314405; doi:10.1186/1471-2431-11-109)
Supplement: Additional file 1 — Hierarchical logistic regression model diagnostics. [file 1471-2431-11-109-S1.DOC]

# Hierarchical logistic regression model diagnostics

| 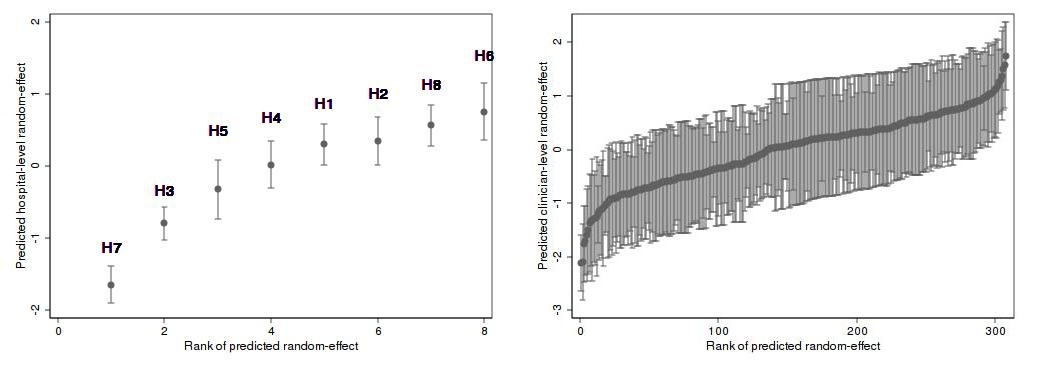 |
| --- |
| *Figure 5: Plots showing distributions of predicted hospital- and clinician-level random-effects. Clinician and hospital-level standard deviations were different from zero, and model-predicted hospital-level and clinician-level random-effects were approximately randomly distributed with a zero mean as expected of this model. This indicates potentially major variation in performance among clinicians and between hospitals* |
